# Proteasome associated function of UCH37 is evolutionarily conserved in *Plasmodium* parasites

**DOI:** 10.1038/s41598-024-80433-y

**Published:** 2024-11-27

**Authors:** Mohsen Hajisadeghian, Annie M. Geiger, Carla Briggs, Cameron Smith, Katerina Artavanis-Tsakonas

**Affiliations:** Department of Pathology, University of Cambridge, Cambridge, UK

**Keywords:** Deubiquitylating enzymes, Ubiquitylation, Parasitic infection

## Abstract

Ubiquitin C-terminal hydrolase 37 (UCH37 also known as UCHL5) is a conserved deubiquitinating enzyme (DUB) with dual roles in proteasomal degradation and chromatin remodeling in humans. Its *Plasmodium falciparum* ortholog, PfUCH37, is unusual in that it possesses both DUB and deneddylating activities. While PfUCH37 is enriched in proteasome preparations, its direct interaction and broader functions in *Plasmodium* remain unclear, particularly given the absence of the chromatin remodeling complex INO80 homologs. This study utilizes transgenic parasites and proteomics to identify PfUCH37-associating proteins. We confirm a direct interaction with the proteasome and demonstrate that the interaction mechanism is evolutionarily conserved. Notably, we discover a divergence in localization compared to the human enzyme and identify novel interacting partners, suggesting alternative functions for PfUCH37 in *Plasmodium*. These findings provide insights into the unique biology of this enzyme in malaria parasites, potentially opening avenues for targeted therapeutic interventions.

## Introduction

UCH37/UCHL5 (PF3D7_1117100), a ubiquitin hydrolase (DUB) conserved across eukaryotes, has been shown to interact with both the proteasome and the INO80 chromatin remodeling complex in human cells^1,2^. This interaction is mediated by the enzyme’s carboxy-terminal autoinhibitory domain binding to a DEUBAD domain in either Adrm1/Rpn13 (a proteasomal subunit) or NFRKB (an INO80 subunit). The binding to Adrm1/Rpn13 activates UCH37^3,4^, while the binding to NFRKB inhibits it^4,5^. These interactions highlight a complex regulatory mechanism controlling this enzyme’s catalytic activity which is essential to cells, since its deletion leads to embryonic lethality in mice^6^, and its dysfunction has been implicated in various cancers^7–9^.

Alongside USP14, another proteasome-associated DUB, UCH37 is known to cleave Lys48 ubiquitin chains off of proteins targeted for proteasomal degradation^10^, a process crucial for maintaining cellular homeostasis. UCH37 has been shown to progressively cleave ubiquitin moieties from the distal end of Lys48-linked chains^10,11^in order to recycle Ub in the cell and allow substrate polypeptides to escape degradation^12,13^. However, its role in INO80-related functions, such as chromatin remodeling and transcriptional regulation, remains unclear. Since ablation of Adrm1/Rpn13- and thereby the interaction of UCH37 with the proteasome- is tolerated by cells, its interaction with INO80 appears to be critical for cell homeostasis^14^.

This role is particularly intriguing when considering UCH37 function in the malaria parasite *Plasmodium falciparum*, where INO80 homologs are absent^15^and deletion of PfUCH37 is possibly lethal as suggested by Piggybac insertion mutagenesis^16^. This raises questions about the potential functional divergence of PfUCH37 from its human counterpart. PfUCH37 possesses both deubiquitinating and deneddylating activities. We have previously shown that its deneddylating activity is dispensable for asexual development^17^ which suggest that its DUB activity could be essential during the blood stage of the parasite’s life cycle. Despite being enriched in *P. falciparum *proteasome preparations^18^, the exact nature of this association and the broader functions of PfUCH37 in *Plasmodium* biology remain undefined.

To investigate these questions, we employed parasite transgenics and proteomics to identify proteins that interact with PfUCH37. We confirmed a direct interaction between PfUCH37 and the proteasome and demonstrated that the mechanism behind this interaction is similar to that observed in human cells. However, we also observed differences in the localization of PfUCH37 compared to the human enzyme and identified putative non-proteasomal interacting partners. These findings suggest that PfUCH37 may have divergent biological roles in *Plasmodium*, different to those in human cells, opening up new avenues for investigating the unique functions of this enzyme in *Plasmodium* and its potential as a target for antimalarial therapies.

## Results

### PfUCH37 is mainly cytoplasmic

**Fig. 1:**
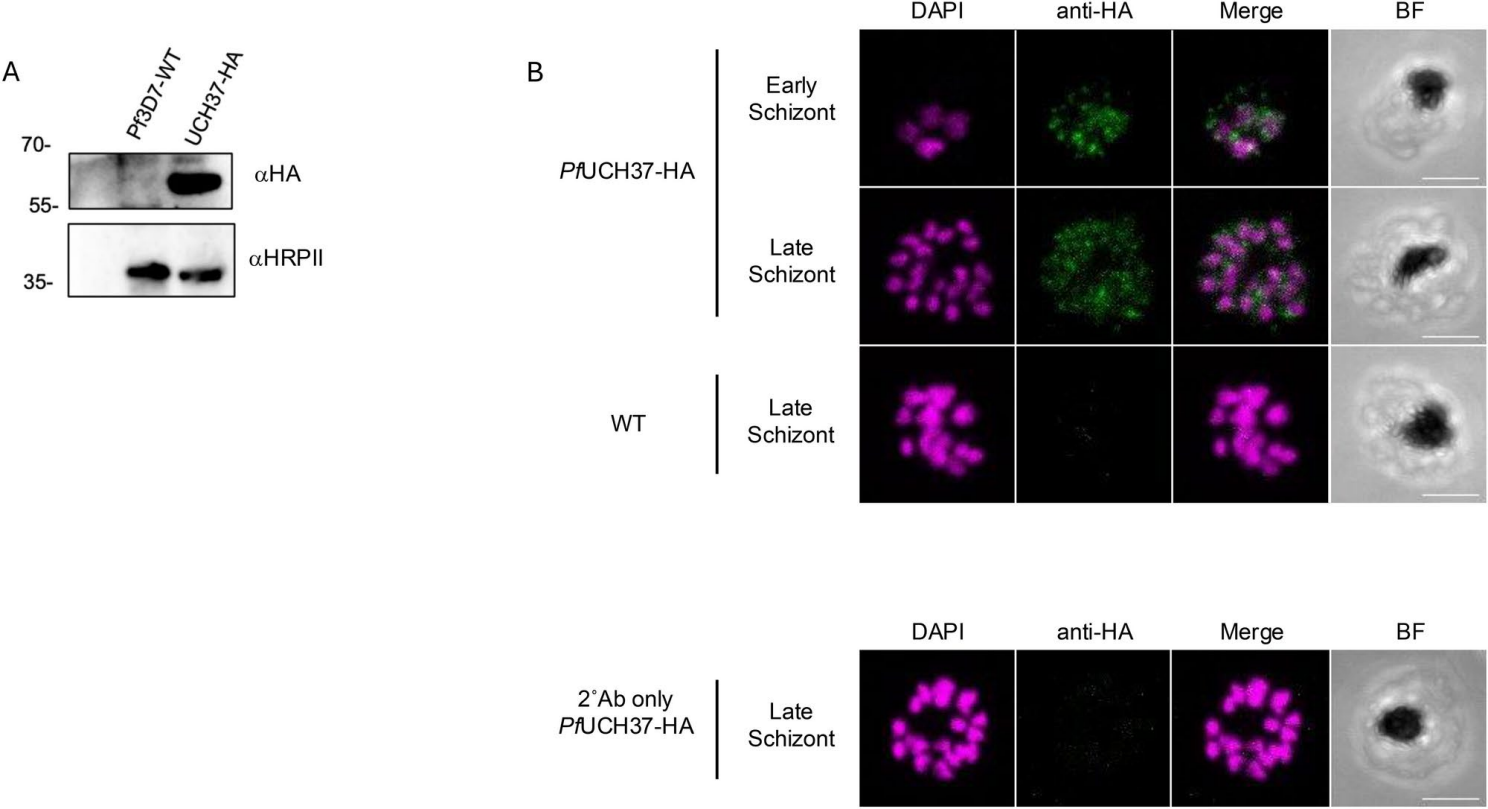
Characterisation of PfUCH37-HA transgenic parasites. (**A)** Anti-HA immunoblot of wild type 3D7 and endogenously HA-tagged PfUCH37 parasite lysate; expected size of PfUCH37-HA at ~ 55 kDa. Detection of HRPII was used as a loading control. (**B)** Anti-HA (green) immunofluorescence assay on endogenously HA-tagged PfUCH37 or wild type 3D7 parasites at mature stages of asexual development. DAPI nuclear stain shown in magenta. Scale bar = 5um. Parasites were imaged by confocal microscopy. Pearson coefficient for colocalization 0.6 ± 0.08 calculated by analysing the HA-signal of 10 randomly-selected schizonts against DAPI. The region of interest used is the entire rectangular panel shown.

To investigate PfUCH37 localisation and protein interactions, we generated a transgenic parasite line wherein the endogenous PfUCH37 gene was tagged with HA on its carboxy-terminus (Supplementary Fig. 1A). Integration was confirmed by PCR (Supplementary Fig. 1B), expression of the tagged protein, and thus proper insertion of the transgene, was confirmed by immunoblot (Fig. 1A) and localisation was probed by immunofluorescence (Fig. 1B). Unlike the distribution of UCH37 in mammalian cells which is mainly nuclear and a bit cytoplasmic^19,20^, PfUCH37 was found to be more widely distributed, without a clear nuclear localization throughout the later stages of asexual development.

### PfUCH37 associates with the proteasome

In humans, UCH37 associates with either the proteasome, via an interaction with the proteasomal subunit Rpn13, or with the chromatin remodelling complex via NFRKB. In both cases, this interaction is mediated through binding of the C-terminus of UCH37 to a DEUBAD (DEUBiquitylase ADaptor) domain within each partner protein but resulting in opposite outcomes. Rpn13 can disrupt UCH37’s inhibitory dimeric structure and potentially its oligomerisation^21^. More importantly, Rpn13 contributes to UCH37 activation by stabilising the substrate-binding conformation, ensuring the active site remains accessible to ubiquitin^1^, whereas NFRKB inhibits UCH37 by obstructing the ubiquitin-binding region and disrupting the active site. This inhibition leads to the sequestration of UCH37 in the nucleus, where it is recruited to the INO80 complex, in turn facilitating ATP-dependent nucleosome remodelling, essential for transcription regulation and DNA repair.

**Fig. 2:**
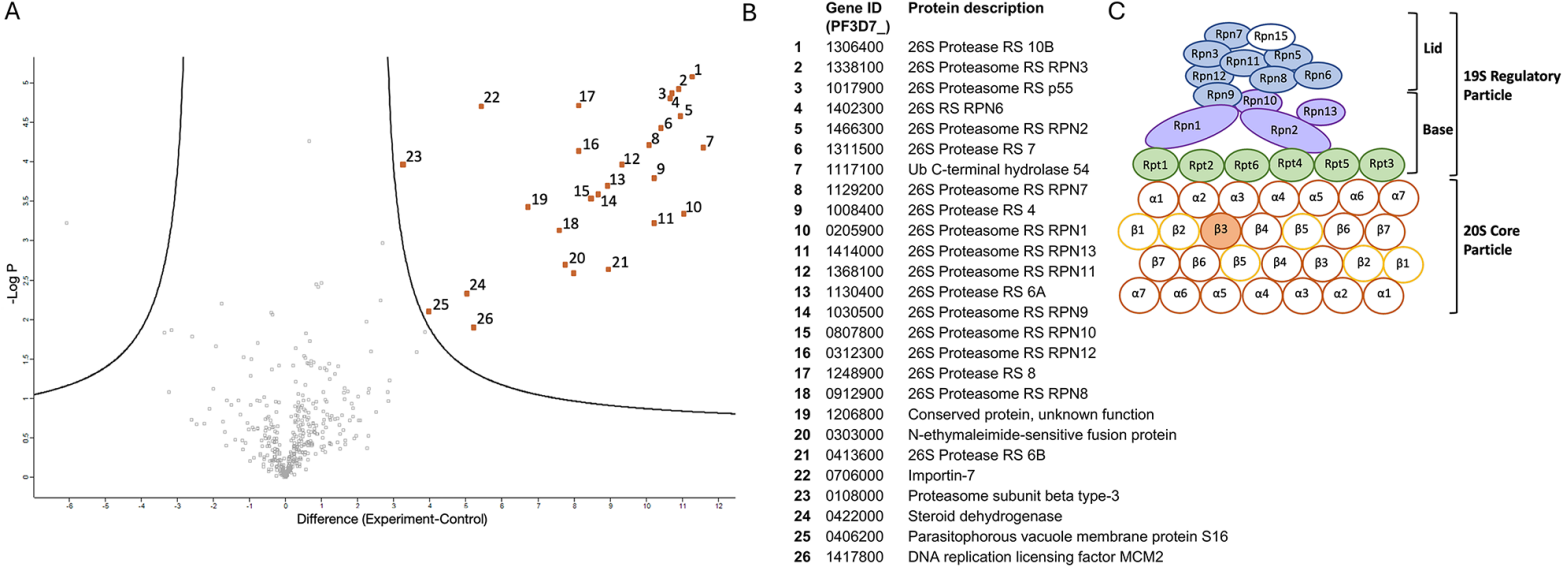
Identification of PfUH37 interacting proteins.(**A**) Volcano plot of processed mass spectrometry data from 3 biological replicates comparing anti-HA co-IP of PfUCH37-HA-expressing parasites with wild type strain 3D7. Numbered points represent hits significantly more enriched through the PfUCH37-HA pulldown. (**B**) List of genes corresponding to the numbered points of the volcano plot listed as Pf3D7_ codes. (**C**) Cartoon schematic of the 19S and Regulatory and 20S Core particles of the proteasome. Coloured components represent proteins that were identified by mass spectrometry.

To investigate whether a similar regulatory mechanism exists in *Plasmodium*, we analysed the proteins co-immunoprecipitated with UCH37 to identify interactors. The subcellular distribution of PfUCH37 alongside the lack of identifiable INO80 homologs would suggest that, in *Plasmodium,* this enzyme lacks the defined nuclear function it has in higher eukaryotes. We captured proteins associating with PfUCH37 through anti-HA immunoprecipitation in wild type 3D7 and PfUCH37-HA-expressing parasites and identified them through LC–MS/MS mass spectrometry. We identified 26 proteins as significant interactors (Fig. 2A), 19 of which were proteasomal subunits (Fig. 2B-C). These were mainly subunits of the 19S regulatory particle lid and base, although one beta subunit of the 20S core particle was pulled down as well. All known subunits of the 19S regulatory particle co- immunoprecipitated with PfUCH37, except for subunit Rpn15. Six other non-proteasomal proteins were also identified; among these were N-ethylmaleimide-sensitive fusion protein and parasitophorous vacuole membrane protein S16, an integral surface membrane protein of sexual stage parasites^22^ which is particularly well-expressed at the onset of gametocytogenesis^23^. A steroid dehydrogenase with putative- 3-hydroxyacyl-CoA dehydrogenase, oxidoreductase, and fatty acid biosynthetic processing activity was also identified^24^as was a putative importin-7 protein involved in GTPase binding and protein import into the nucleus as a nuclear transport receptor or through association with the importin-beta subunit KPNB1^25^. A conserved *Plasmodium *protein of unknown function (PF3D7_1206800) and the DNA replication licensing factor MCM2 were the final two non-proteasomal proteins to be identified. Interestingly, MCM2 was also found to coimmunoprecipitate with human UCH37 and proteasome components and was highlighted as a potential substrate of this DUB^26^.

**Fig. 3:**
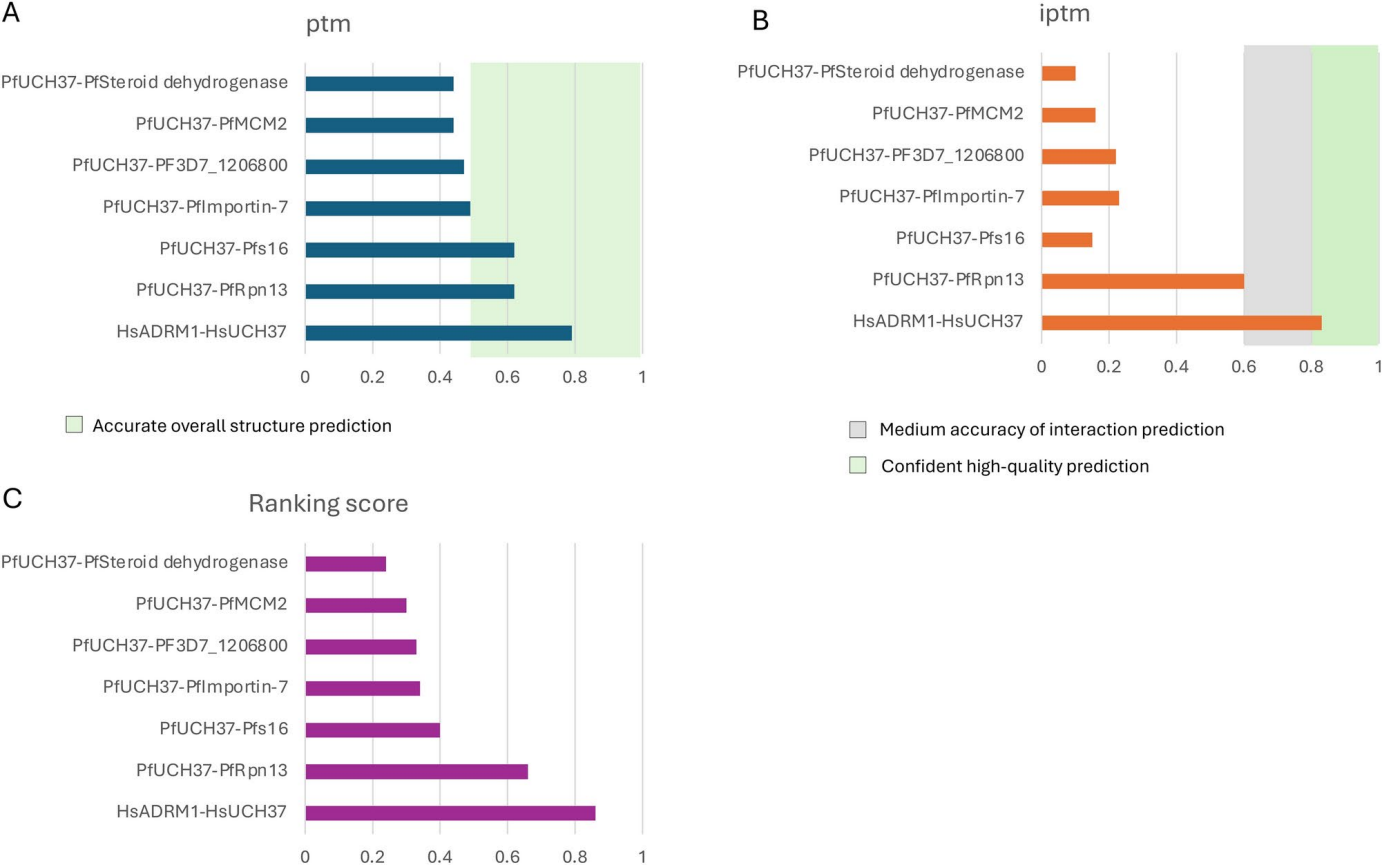
Interaction predictions for PfUCH37 and mass spec hits. Alphafold3 was used to predict the likelihood of a direct interaction between PfUCH37 and the top proteomic hits. (**A)** The predicted template modelling score (pTM) measures the likelihood that the overall predicted fold for the complex might resemble the true structure, with a value above 0.5 representing good confidence. (**B)** The interface predicted template modeling (ipTM) score assesses the accuracy of the predicted relative positions of the subunits within the complex. Values above 0.8 indicate confident high-quality predictions, while values below 0.6 suggest likely failed predictions. Scores between 0.6 and 0.8 fall into a gray zone where predictions may be correct or incorrect. (**C) **Interface metrics for protein–protein interactions are ranked based on a bespoke ipTM aggregate score representing the two chains in the interface. The interface ranking was conducted using the method detailed in^27^.

Protein sequence and structural homology searches did not identify DEUBAD domains in any putative interactors. Nevertheless, we questioned whether the non-proteasomal mass spectrometry hits could be bona fide partner proteins and used AlphaFold 3^27^to model the likelihood of their interaction with PfUCH37. The interaction of human UCH37 and ADRM1 was used as a reference for the accuracy of the prediction^5^,yielding a pTM score of 0.79 and ipTM of 0.83. The interaction scores for the rest of the proteomic hits were well below this score (Fig. 3) indicating that they either interact with PfUCH37 indirectly, or were captured non-specifically. The only one yielding an appreciably confident score was the interaction of PfUCH37 with PfRpn13. Although the prediction for a PfUCH37 and PfS16 complex resulted in a relatively high pTM score of 0.62, the ipTM score suggested a lack of confidence in the predicted interaction.

### PfUCH37 and PfRpn13 interact in a similar manner to the orthologous human proteins

Since the interaction between PfUCH37 and the proteasome was most likely given our analysis and the defined interaction of the orthologous proteins in mammalian cells, we set out to biochemically validate this result.

**Fig. 4:**
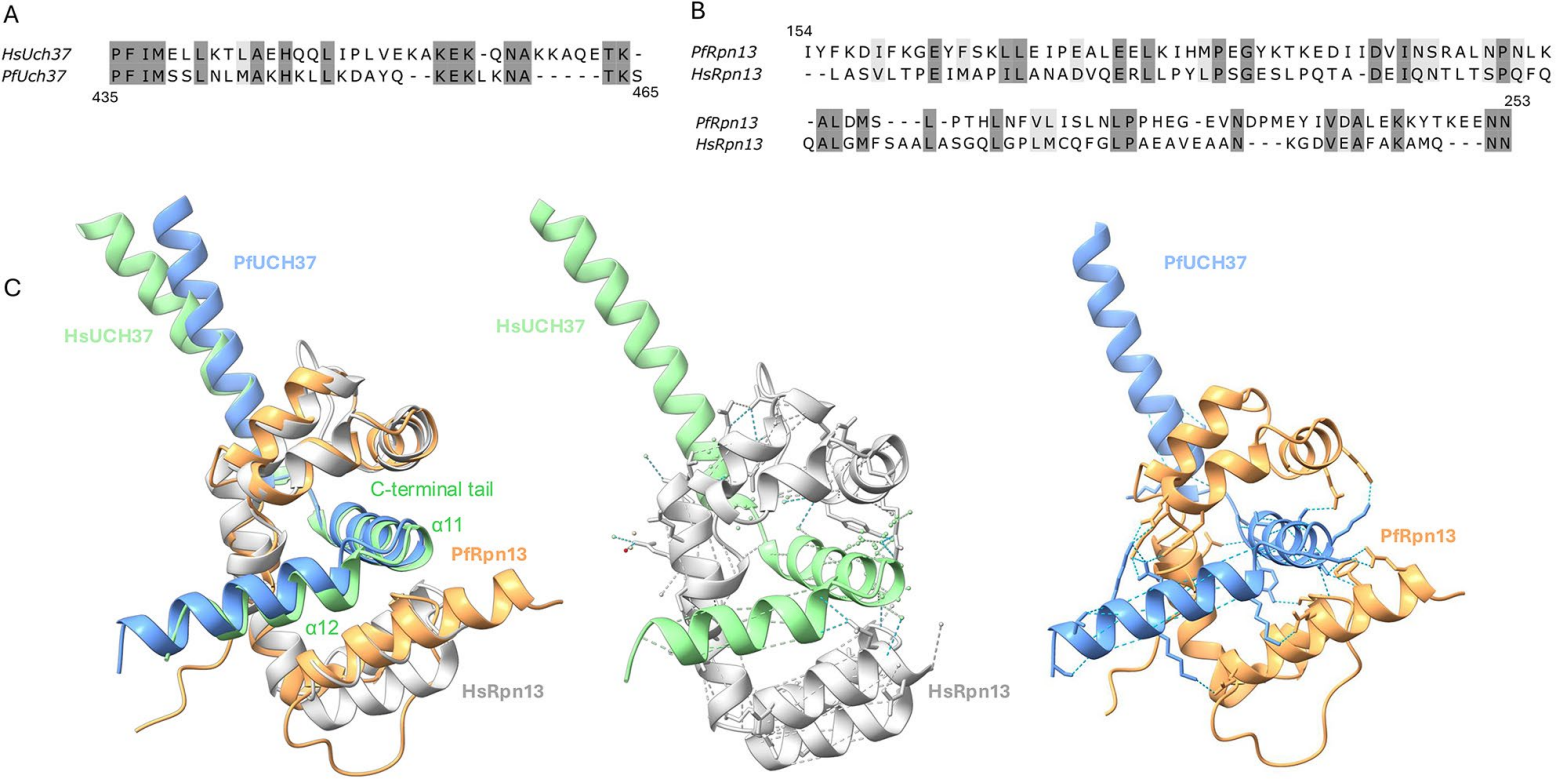
Structural modelling of the interaction between PfUCH37 and PfRpn13. (**A)** Alignment of the C-terminal tail of human UCH37 with that of *Plasmodium falciparum*. (**B)** Alignment of the DEUBAD domains of Rpn13 in *Plasmodium falciparum* and humans. Conserved residues are highlighted in dark gray, while conservative replacements are shown in light gray. (**C)** Structural analysis of the protein-protein interaction (PPI) interface between the UCH37 C-terminal tail and the Rpn13 DEUBAD domain in humans and *Plasmodium falciparum*. **Left:** Superimposition of the crystal structure of the human UCH37 C-terminal tail (green, residues 265–320) and the human Rpn13 DEUBAD domain (gray, residues 287–384) complex (PDB: 4UEL) with the AlphaFold predicted-structure of the *Plasmodium falciparum* UCH37 C-terminal tail (blue, residues 409–465) and Rpn13 DEUBAD domain (orange, residues 150–253). **Middle:** Interactions of human UCH37/Rpn13 complex interface. **Right:** Interactions of the *Plasmodium falciparum* UCH37/Rpn13 complex interface. Light green dotted lines indicate hydrogen bonds.

In human and mouse cells, UCH37 is known to interact with the proteasome by binding Adrm1/Rpn13. Specifically, The carboxy-terminal regions of both Rpn13 and UCH37, which contain KEKE motifs, play a crucial role in their physical interaction and assembly of the 26S proteasome. A KEKE motif is defined as a sequence longer than 12 amino acids, devoid of W, Y, F, or P residues, composed of more than 60% lysine (K) and glutamic acid/aspartic acid (E/D), and lacking five consecutive residues that are positively (K, R, H) or negatively (D, E) charged. These motifs are recognized for their role in mediating protein–protein interactions^28^. We observed that PfUCH37 contains a KE-rich region at its C-terminal end (Fig. 4A). Although this region does not fit the previously defined KEKE motif, it is abundant in lysine and glutamic acid residues. Therefore, we used Alphafold 3^27^ to dock models of these two proteins and determine the most likely conformation. The protein–protein interaction confidence metrics (ipTM = 0.92 pTM = 0.78) were high (Supplementary Table1) and the proteins do appear likely to interact via their KEKE domains (Fig. 4C, right panel). Overlaying the predicted *Plasmodium* complex interface with the solved crystal structure of the human proteins indicates that the interactions occur in a very similar manner (Fig. 4C, left panel). However, amino acid sequence alignment of the interaction domains of each protein revealed a number of differences (Fig. 4A-B). The structural conservation of the DEUBAD domains in PfRpn13 and HsRpn13 is evident in their comparable binding modes to UCH37. In both species, the core DEUBAD domains interact with the C-terminal tail of UCH37 (Fig. 4C). In human UCH37, the amphipathic helix α11 in the C-terminal tail is clasped by the DEUBAD domains and further stabilised by helix α12, forming an extensive hydrophobic interface^5^. AlphaFold predicts a similar interaction for PfUCH37. Thus, we hypothesised that the binding of the *Plasmodium *DEUBAD domain also requires these helices, as a human UCH37 variant lacking them (UCH-L5Δα11–12) fails to interact with Rpn13^5^. In HsUCH37, GLU300 forms critical hydrogen bonds with ARG309 and TYR313 in Rpn13. However, in PfUCH37, this residue is replaced by SER440, which cannot form similar bonds with the DEUBAD domain. To identify the amino acids involved in the protein–protein interaction and compare the PPI interface between these two species, we used PDBePISA 2007^29^. Complex stability in biological systems is influenced by key physicochemical properties, such as free energy of formation, interface area, hydrogen bonds, salt bridges, and hydrophobic interactions. The PISA tool combines these factors to analyse a structure and predict the potential stability of a macromolecular complex. The results, summarised in Supplementary Table 2, revealed differences in both the interacting residues and the types of bonds formed, elements that could be targeted to disrupt the PfUCH37-Rpn13 interaction. Nonetheless, further structural analysis is necessary to validate the accuracy of the AlphaFold predictions.

### The interaction between PfUCH37 and PfRpn13 requires an intact KEKE domain

**Fig. 5:**
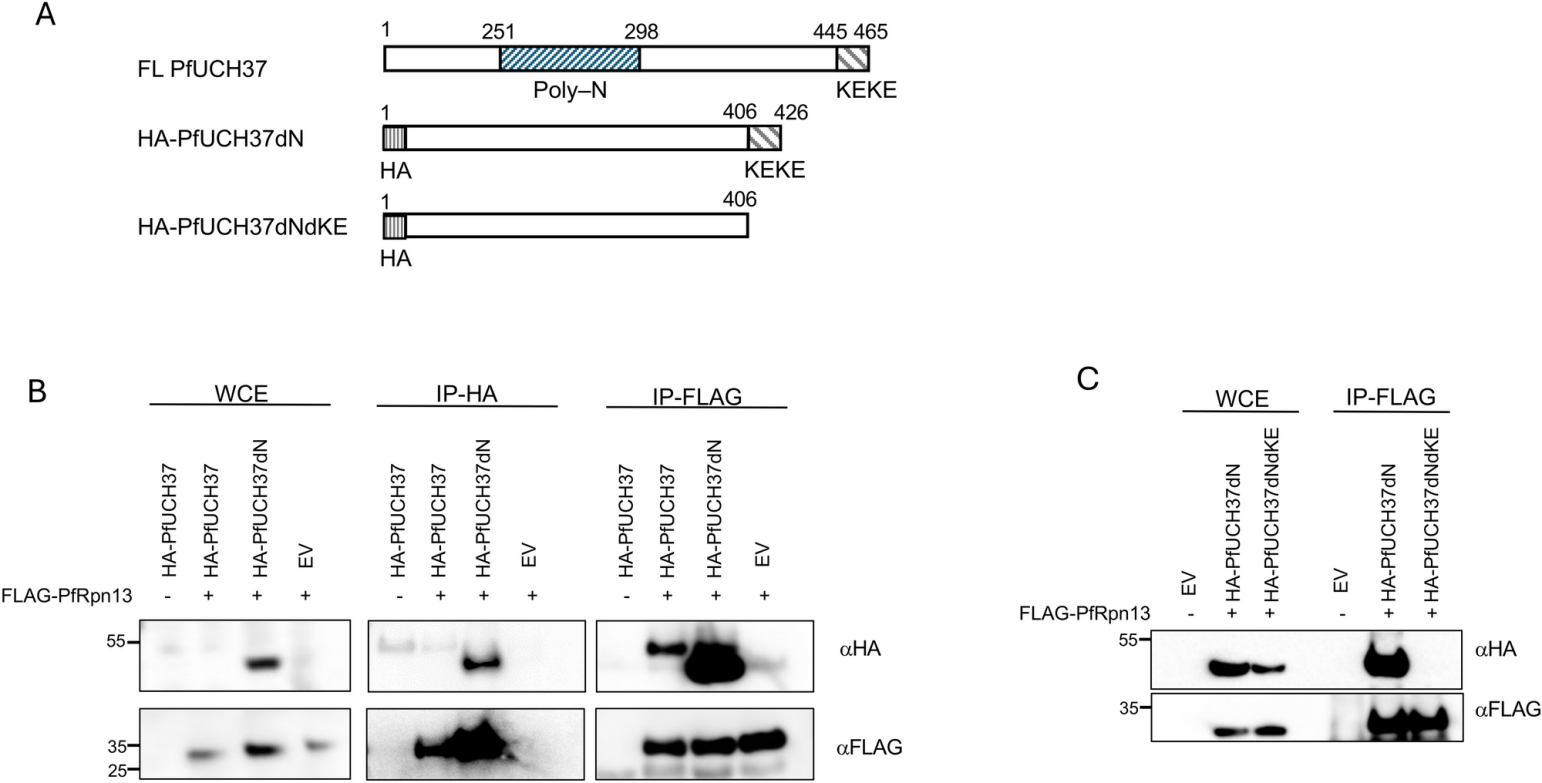
Interaction of PfUCH37 with PfRpn13.** (A)** Schematic of PfUCH37 domains and truncation proteins generated for interaction studies. (**B)** HEK293 cells co-expressing HA-tagged PfUCH37 (wild type or poly-asparagine region deleted (dN)) and FLAG-tagged PfRpn13 were subjected to affinity purification using either anti-FLAG or anti-HA magnetic beads. Cell lysates expressing only PfUCH37 or only PfRpn13 served as negative controls for each immunoprecipitation experiment. (**C)** The experiment was repeated using a C-terminal KEKE motif (dKEKE) deletion mutant to assess its ability to interact with PfRpn13. WCE: whole cell extract, EV: empty vector, IP: immunoprecipitation. Expected MW of PfRpn13 is 29 kDa and of PfUCH37 is 54 kDa.

With convincing in silico evidence that the association of PfUCH37 with the proteasome is mediated via a direct interaction with the PfRpn13 subunit, we next set out to confirm this interaction in vitro. We generated expression constructs of HA-tagged PfUCH37 and FLAG-tagged PfRpn13, codon-optimised for expression in human cells. Like many *P. falciparum* proteins, PfUCH37 contains an intrinsically disordered, poly-asparagine-rich region (between amino acid positions 250–299). We anticipated that the presence of the poly-asparagine-rich region might adversely affect recombinant expression of the protein, thus we also generated an HA-tagged construct lacking this region (PfUCH37dN) (Fig. 5A). Previous studies have demonstrated the dispensability of poly-asparagine repeats for protein function, and we have shown this to be true for PfUCH37 where deletion of this region does not affect the folding of the catalytic domain, nor does it negatively impact enzyme activity against either Ub-AMC or Nedd8-AMC^17^. Moreover, AlphaFold 3 structure and protein–protein interaction prediction showed the fold of the PfUCH37dN mutant is identical to that of the full length enzyme, and that both the C-terminal KEKE motif and the catalytic cysteine of PfUCH37dN are accessible for interaction with Rpn13 and ubiquitin, respectively (Supplementary Fig. 2).

These constructs were co-transfected into HEK293 cells and the interaction between PfUCH37 and PfRpn13, initially identified through mass spectrometry, was validated using an IP-western blot approach. As expected, the expression of wild-type UCH37 was very low in HEK293 cells whereas the poly-asparagine deletion (UCH37dN) significantly improved expression (Fig. 5B). Nevertheless, IP of both full-length and UCH37dN proteins co-precipitated PfRpn13 as demonstrated by anti-FLAG blot. This interaction was further validated by performing the reciprocal experiment, IP of PfRpn13 followed by anti-HA blot to detect PfUCH37 (Fig. 5B).

To test whether this interaction is dependent on the C-terminal KE-rich region of PfUCH37, we generated a truncation construct missing both the poly-asparagine repeat and the last 20 amino acids aligning with amino acids 313–329 of the human enzyme known to be critical for binding Rpn13 (Fig. 5A). As suspected, deletion of this C-terminal portion entirely ablated the ability of UCH37 to co-precipitate with PfRpn13 (Fig. 5C).

### Association with PfRpn13 increases catalytic activity of PfUCH37

PfUCH37 is thought to liberate the proteasome from difficult to process ubiquitinated substrates by trimming Lys48-linked Ub chains from their distal end^11,30^. As such, its enzymatic action positively regulates proteasomal degradation by preventing proteasome stalling, and also allows recycling of ubiquitin by cleaving unanchored Lys48-linked polyUb chains. Generally, UCH-family DUBs are known to release only small adducts or unfolded polypeptides from the C-terminus of ubiquitin; they do not hydrolyze protein conjugates such as di-ubiquitin. Several Human UCHs such as UCH-L1, UCH-L3, UCH-L5 and Yeast Yuh1 exhibit this characteristic^3,31–33^. Specifically, previous studies have shown that UCH37 alone, or in the UCH37-Adrm1 and UCH37-Adrm1-S1 complexes, also fails to hydrolyze significant amounts of the Lys48-linked di-ubiquitin. We attempted to determine the linkage-cleavage specificity of PfUCH37 by testing its ability to cleave a panel of di-Ub linkage variants (prepared as described^34^by Gerbrand van der Heden van Noort), however, as seen for its human counterpart^3^, the enzyme was unable to hydrolyse di-Ub, in the presence or absence of PfRpn13 (Supplementary Fig. 3).

**Fig. 6:**
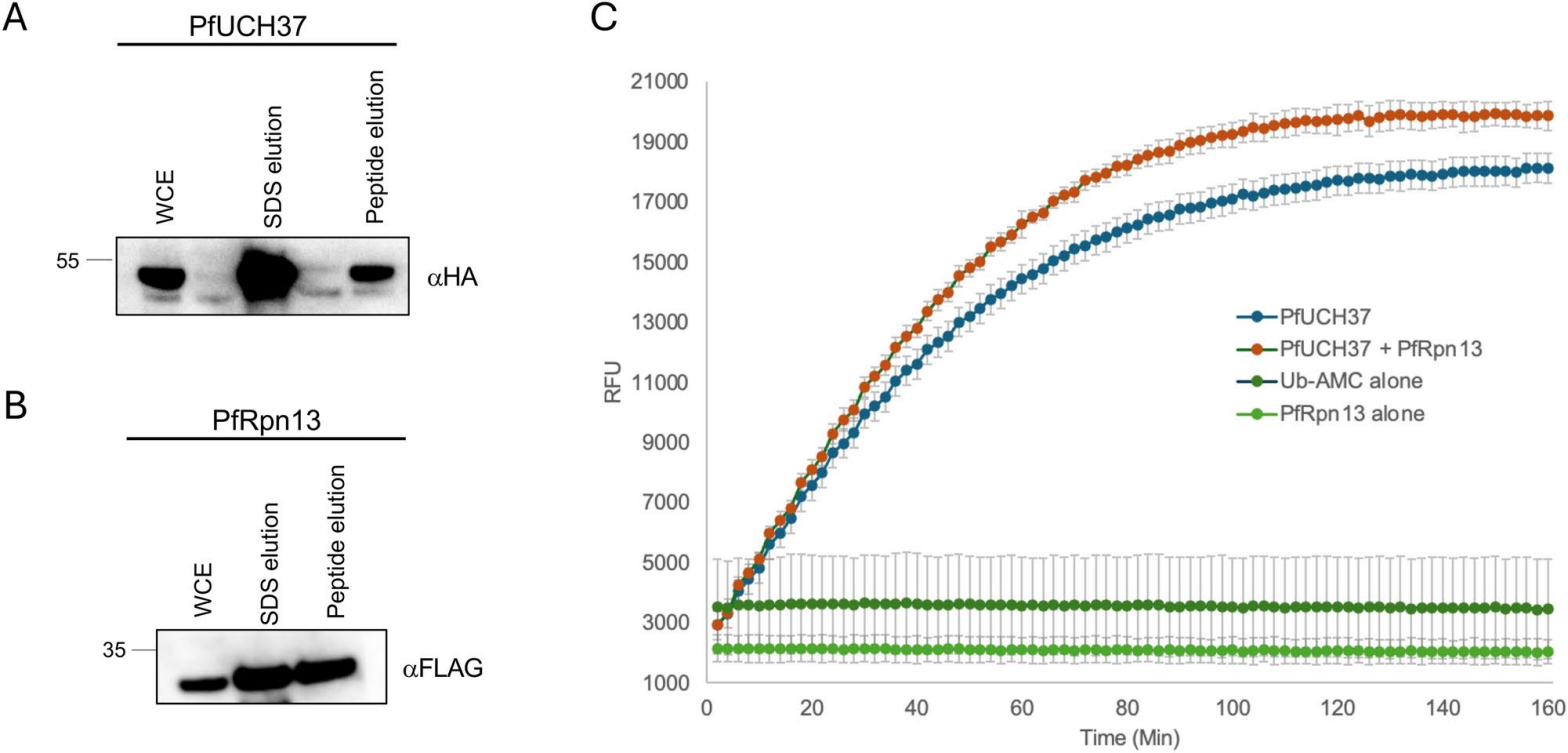
PfUCH37 Ub-AMC activity assay in presence and absence of PfRpn13 . Western blots showing (**A)** purified HA-tagged PfUCH37dN and (**B)** FLAG-tagged PfRpn13 proteins following mammalian expression, purification (anti-HA or anti-FLAG resin), and elution by peptide (HA or 3xFLAG) or SDS. WCE: whole cell extract. (**C)** Assay measuring the enzymatic cleavage of Ub-AMC substrate by PfUCH37dN in the presence or absence of PfRpn13. Cleavage was measured by fluorescence output (in relative fluorescence units, RFU) every two minutes for 160 min. Each measurement was made in triplicate and error bars represent standard deviation.

By binding the C-terminus of UCH37, Rpn13 is able to pull away its autoinhibitory tail and enable access to the enzyme’s active site. As such, UCH37 is more catalytically active in the presence of Rpn13 or whole proteasomes^1,3^. We tested whether this was also the case for PfUCH37 by subjecting it to a Ub-amido-methyl-coumarin (AMC) activity assay. In this assay, enzymes able to hydrolyse ubiquitin will engage with the substrate and cleave the AMC which will fluoresce when released. Fluorescence will accumulate and can be measured in real time, revealing the DUB enzyme’s kinetics. HA-PfUCH37dN and FLAG-PfRpn13 were expressed in 293 cells and purified via capture with anti-HA and anti-FLAG resin, respectively followed by peptide elution. Presence of each protein was validated by immunoblot (Fig. 6A) and quantified by BCA. The activity of PfUCH37dN was assessed by adding 10 µL of 250 nM enzyme to 10 µL of 250 nM Ub-AMC substrate in reaction buffer. The reactions were conducted in the presence of 10 µL of Rpn13 at a 1:1 molar ratio or with 10 µL of reaction buffer, resulting in a final reaction volume of 30 µL.Fluorescence measurements were taken every minute for 3 h until all substrate was depleted, as judged by a plateau in fluorescence emission. Similarly to what has been observed for the human enzyme, PfUCH37 activity was higher in the presence of PfRpn13, with Ub-AMC substrate becoming depleted more quickly (Fig. 6B). Although PfRpn13 does not enhance Uch37 cleavage of di-ubiquitin, it markedly triggers Uch37-catalysed hydrolysis of ubiquitin-AMC (Fig. 6C). Therefore, in *Plasmodium*, Rpn13 not only recruits UCH37 to the proteasome but also amplifies its deubiquitination activity.

## Discussion

Our study uncovers a direct interaction between the *Plasmodium falciparum *ubiquitin C-terminal hydrolase, PfUCH37, and the proteasome subunit PfRpn13, mediated through the enzyme’s carboxy-terminal KEKE motif. Mass spectrometry and in silico docking analysis did not identify any other co-immunoprecipitating proteins as being likely direct interactors of this enzyme which diverges from what has been shown in the mammalian system. Human UCH37 interacts with both the proteasome, via Rpn13, and the INO80 complex in the nucleus via NFRKB. Its association with INO80 keeps UCH37 in an inactive conformation and also appears to mediate the essential function of this DUB in mammalian cells, as Rpn13 can be deleted with no adverse effect on cell viability or proteasome function^14^. The apparent absence of INO80 subunits in *Plasmodium* or other direct interactors beyond PfRpn13 points to the proteasome-associated role of PfUCH37 as its primary function in the parasite.

This disparity raises the possibility that the unique life cycle of the parasite necessitates a different mode of regulation for its UCH37 enzyme. Unlike in humans, where UCH37 is held in an inactive state until recruited to the proteasome, the rapid growth and development of *Plasmodium* may not require this level of regulatory control. Moreover, whereas deletion of mammalian UCH37 is embryonic lethal, its essentiality to *Plasmodium* is more ambiguous. Disruption of this gene in different *Plasmodium* species results in a spectrum of phenotypes. Piggybac mutagenesis in *P. falciparum* suggests it is essential whereas this same approach in *P. knowlesi* points to dispensability. Disruption in the mouse model *P. berghei* would suggest something in between: mutant parasites are alive but grow significantly slower than wild-type. Although direct confirmation of essentiality requires generation of a conditional knockout in *P. falciparum*, it is highly probable that disrupting UCH37 function in malaria parasites would lead to some form of cellular stress, if not during asexual replication then possibly at other stages of the lifecycle.

Similarly to what has been observed in mammalian cells, we demonstrate that the interaction with the proteasome, through binding of the PfRpn13 subunit, significantly enhances the catalytic activity of PfUCH37. To determine whether this increase in catalytic activity is critical for proper PfUCH37 function in the parasite, a transgenic line with a conditional KEKE deletion would be the ideal approach. As a preliminary step, we attempted to episomally overexpress a non-conditional KEKE deletion mutant. However, three separate transfection attempts failed, while parasites transfected in parallel with the full-length control construct consistently survived. This result suggests that overexpression of the KEKE deletion mutant may be creating a dominant negative effect, suggesting that PfRpn13-mediated activation of PfUCH37 is indeed important for parasite viability.

Consequently, this interaction emerges as a compelling target for the development of novel protein–protein interaction (PPI) inhibitors. A comparison of the active sites of the human and *Plasmodium *enzymes reveals differences that could theoretically be exploited for targeted drug development. Additionally, the conformational differences observed in the interaction interfaces between the enzymes and their respective Rpn13 subunits offer further potential for the design of selective inhibitors, as we have recently demonstrated for the related PfUCHL3 DUB^35^. Moreover, the high conservation of UCH37 across different *Plasmodium* species strengthens its potential as a target for broad-spectrum antimalarials.

In *Plasmodium*, PfUCH37 also exhibits the unique characteristic of dual Ub and Nedd8 specificity, a feature that further distinguishes it from the mammalian enzyme. Although we did not directly assess whether Nedd8 hydrolysis is also increased by PfUCH37 interaction with PfRpn13, it is plausible that increased access to the enzyme’s active site would enhance Nedd8 cleavage as well. While we have previously shown that Nedd8 activity is dispensable for asexual parasites^17^, a recent study disrupting neddylation in *P. berghei *across asexual and sexual life cycle stages determined that Nedd8 is essential for ookinete formation^36^. This observation suggests a potential role for PfUCH37 and its Nedd8 hydrolysis in mosquito stages.

In conclusion, our findings provide foundational evidence on the proteasomal role of PfUCH37, its protein associations and how these affect its catalytic activity. Further investigation into the function of this enzyme in *Plasmodium* is crucial for understanding its unique biology and exploring its potential as a novel antimalarial target.

## Materials and methods

### Growth and maintenance of parasites

*Plasmodium falciparum *3D7 parasites were maintained in human erythrocyte culture at 4% hematocrit as previously described^37^. Cultures were synchronised through treatment with 5% sorbitol for 5 min at room temperature before being washed twice in incomplete RPMI and returned to standard culture conditions. At 8% parasitemia of late trophozoite/schizont stages, parasites were released from erythrocytes using 0.15% saponin, washed twice in PBS, and erythrocyte-free parasites were pelleted at 800 g before being snap frozen and stored at −80C.

### Generation of transgenic parasite lines

HA-tagging of the PfUCH37 gene was accomplished by single crossover homologous recombination. A 1231-bp region of homology (nucleotides 166–1397) of the single exon ORF of Pf3D7_1117100 was amplified by PCR from *P. falciparum *genomic DNA and cloned with a C-terminal HA tag followed by the 3’ UTR of hsp86 into pCAM-BSD^38^. Transfection was performed by electroporating sorbitol-synchronized, ring-stage parasites in the presence of 100ug of plasmid DNA and at 5 h post transfection, blasticidin (Sigma) was added to a final concentration of 2.5 mg/ml to select for transformed parasites. Blasticidin-resistant parasites appeared 3 to 4 weeks post transfection.To enrich for transfectants, parasites were subjected to blasticidin cycling by culturing 3 weeks without blasticidin followed by 1 week with blasticidin. This cycling was repeated twice. Integration was confirmed by PCR.

### Co-Immunoprecipitation

Parasite pellets derived from 70 ml cultures of *P. falciparum* at 4% haematocrit and 8% trophozoite/schizont parasitemia were each lysed in 1 ml of lysis buffer (50 mM HEPES, 150 mM NaCl, 1% TritonX100, protease inhibitor, 1 mM PMSF, pH 7.5). Lysed pellets were freeze-thawed three times on dry ice before supernatant clarification through centrifugation. Protein concentration was determined through BCA assay, and samples were standardized to 0.692 mg of protein each. Samples were pre-cleared with Neutravidin beads equilibrated in wash buffer (50 mM HEPES, 150 mM NaCl, 0.1% TritonX100, protease inhibitor, 1 mM PMSF, pH 7.5) for one hour, rotating at 4 °C and then transferred to a new tube containing HA-conjugated resin (equilibrated in wash buffer) and incubated for 2.5 h rotating at 4 °C. Resin was washed three times in wash buffer before captured proteins were eluted by the addition of two consecutive treatments with 25 μl of HA-peptide in elution buffer (1 mg/ml HA peptide, 15 mM Tris pH 7.4, 150 mM NaCl, 0.1% SDS, 0.5% NP-40). Samples were run in triplicate.

### Mass spectrometry and data processing

Mass spectrometry was performed at the Cambridge Centre for Proteomics. SDS-PAGE separated protein samples were de-stained, reduced with DTT, alkylated, and digested with trypsin. The digestion reaction supernatant was loaded onto an autosampler for LC–MS/MS analysis using a Dionex Ultimate 3000 RSLC nanoUPLC and Q Exactive Orbitrap mass spectrometer. The peptides were separated by reverse-phase chromatography and loaded onto a pre-column, then eluted from the pre-column to the analytical column. An Easy-spray source was used to spray the LC eluant into the mass spectrometer, and mass to charge values were measured for the eluted ions. The MS/MS data collected was searched against common contaminants, and significant peptide values were recorded.

Label-free quantification (LFQ) was conducted using MaxQuant with the proteome file from the PlasmoDB database (The Plasmodium Genome Database Collaborative, 2001) and statistically analysed using Perseus (Perseus, 2016). First, values labeled as “Contaminant”, “Reverse”, and “Only identified by site” were filtered out of the dataset. The data was logarithmised by a log base two formula. The samples were grouped into categorical annotation rows and split into test and control groups. A two-sample Student’s t- test was performed to identify interacting proteins. A volcano plot was generated from these results, with conditions of a minimal fold change (s0) value of two and a False Discovery Rate (FDR) value of 0.01.

### Immunofluorescence Assay (IFA)

The IFA protocol was adapted from Tonkin et al., 2004^39^. Briefly, 1.5 ml of an asynchronous *P. falciparum *culture of 5% parasitemia was centrifuged at 450xg for one minute and washed in 1xPBS. The pellet of 50–100μl was resuspended in 1 ml of fixation solution (4% paraformaldehyde in 1xPBS). The solution was incubated for 10 min, rotating, at RT. The fixation solution was washed off with 2 × 1 ml of PBS (centrifuged at 3000 rpm for one minute). The cells were permeabilised with 1 ml of 0.15% Triton X-100 in PBS, rotating at RT for fifteen minutes. The permeabilisation solution was washed off as previously described, and the cells were resuspended in 0.5 ml of blocking solution (1% BSA in PBS) for 30 min at RT. This solution was replaced with 0.15 ml of primary antibody (rat 3F10 anti-HA diluted 1:1000 in blocking solution) and incubated for one hour, rotating, at RT. The primary antibody was removed by 3 × 1 ml washes with PBS, rotating for five minutes. 0.25 ml of secondary antibody (Thermo Fisher Scientific anti-rat Alexa Fluor 488 diluted 1:1000 in blocking solution) was added and incubated for one hour, rotating, at RT. The secondary antibody was washed off with 2 × 1 mL PBS, then incubated with 2 μg/ml DAPI . This was followed by washes of 2xPBS. Primary and secondary antibody control samples were included, where each sample was treated with only the respective antibodies. The cell pellet was resuspended in its original volume of PBS. 10 μl of this was smeared on a slide and dried. 1 drop of ProLong Diamond Antifade Mountant was applied to the edge of the slide, and covered with a coverslip. The slides were cured overnight in the dark at RT and imaged on a Zeiss LSM700 confocal microscope. Image and statistical analysis were completed in ImageJ^40^.

### Immunoblot

20 μl of sample lysates prepared as described above were separated by SDS-PAGE on a 4–12% Bis–Tris gel at 180 V for 60 min in 1xMOPS buffer. PVDF membrane was activated in methanol and equilibrated in 1 × transfer buffer (25 mM Tris, 192 mM glycine, 10% methanol) and proteins were transferred to the membrane using a semi-dry apparatus at a constant 10 V for one hour. The membrane was blocked overnight in 5% skim milk in 1xTBST (Tris-buffered saline, 0.1% Tween detergent) at 4 °C shaking, or at RT for one hour. Anti-HA antibody (Rat, monocolonal, 11,867,423,001,Roche) or anti-FLAG antibody (Rabbit, F7425, Merck ) was added 1:2000 to 5 ml of 5% skim milk in 1xTBST and incubated, rotating at RT for Two hours. The blot was washed three times for 5 min with 1xTBST and incubated in rat or rabbit secondary antibody (1:4000) for one hour at room temperature, then developed using ECL substrate ( SuperSignal™ West Pico PLUS Chemiluminescent Substrate, 34,580, Thermo Fisher Scientific) before visualisation on an Azure c500 imager.

### Ub-AMC activity assay

Activity of PfUCH37 (1 nM) in the presence or absence of PfRpn13 (1 nM) was tested by Ub-AMC (Boston Biochem) assays as described previously^17^. Protein concentrations were measured and normalised by BCA assay (Pierce), and reactions were initiated by addition of Ub-AMC substrate (125 nM) at the indicated concentrations. Fluorescence was measured in relative fluorescence units (RFU) on a BMG FLUOstar Omega plate reader.

### In-silico analysis

For sequence alignment and residue conservation analysis, we utilised Clustal Omega^41^and JALView^42^. Protein modelling was performed using AlphaFold3, a deep-learning-based modelling method^27^. Interaction surface determination, for both solved and predicted structures, was conducted using the PISA Server^29^. Protein figures were generated with UCSF ChimeraX^43^, and superpositions were executed using the RCSB PDB Pairwise Structure Alignment tool^44^.

## Supplementary Information

Supplementary Information 1.

Supplementary Information 2.

Supplementary Information 3.

## Data Availability

The mass spectrometry proteomics data have been deposited to the ProteomeXchange Consortium via the PRIDE partner repository with the dataset identifier PXD053953.
