# Supplementary material for: Proteasome associated function of UCH37 is evolutionarily conserved in Plasmodium parasites
Source: Sci Rep. 2024 Nov 27;14:29428. doi: 10.1038/s41598-024-80433-y (PMC11603131; doi:10.1038/s41598-024-80433-y)
Supplement: Supplementary file 1 — Supplementary Information 1. [file 41598_2024_80433_MOESM1_ESM.pdf]

Supplementary Figure 1

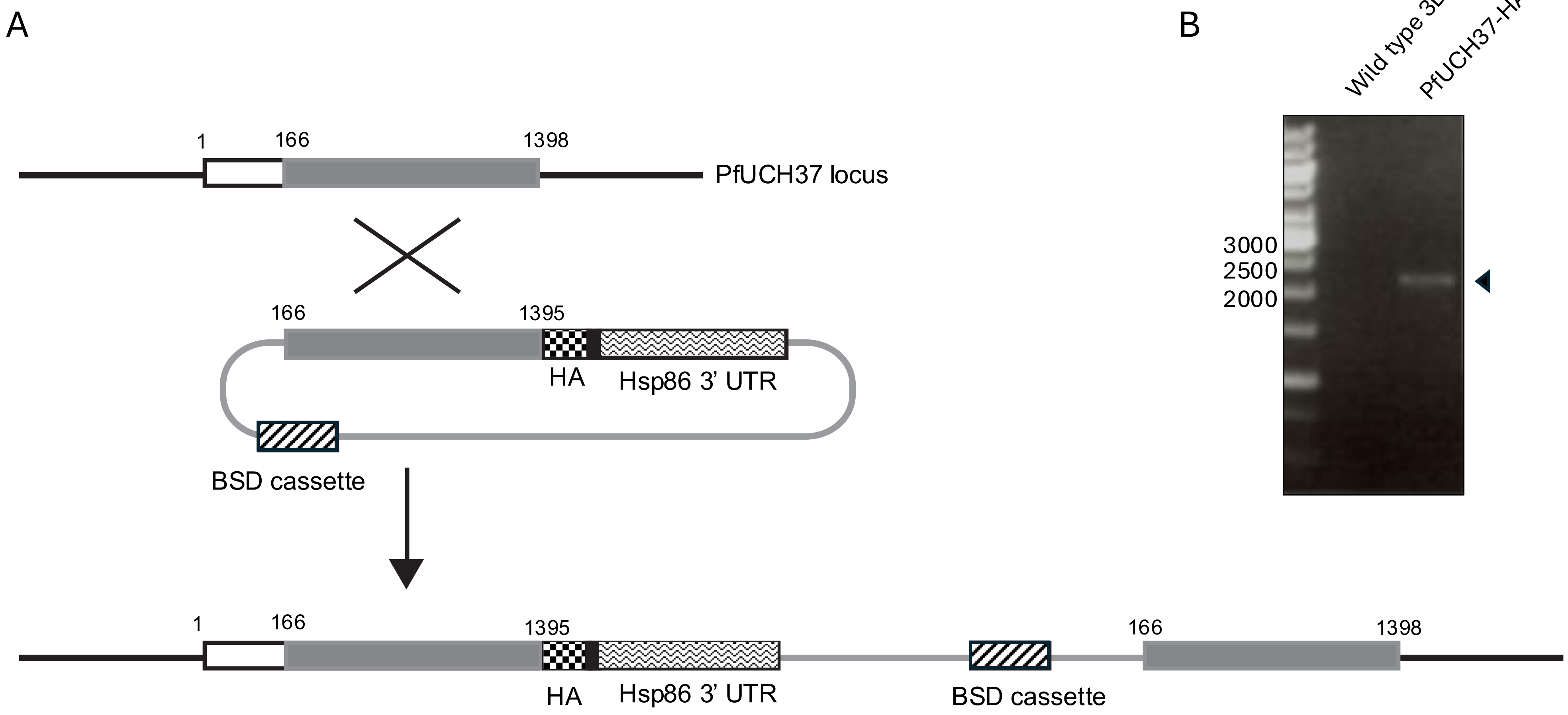

Supplementary Figure 1. Generation of PfUCH37-HA transgenic parasites. A) Schematic representation of HA-tagging of endogenous PfUCH37 gene via single crossover homologous recombination. A 1229-bp region of homology (nucleotides 166-1395) of the single exon ORF of Pf3D7_1117100 was cloned with a C-terminal HA tag followed by a short linker (shown in black) and the 3' UTR of hsp86 into the pCAM-BSD plasmid, containing a blasticidin resistance cassette. **B)** PCR confirmation of successful integration using a primer annealing to the very beginning of the PfUCH37 gene (absent in the transfection plasmid) and the end of the hsp86 3' UTR. No integration would not be expected to produce a product whereas successful integration would be expected to produce a 2.3Kb fr agment.

Supplementary Figure 2

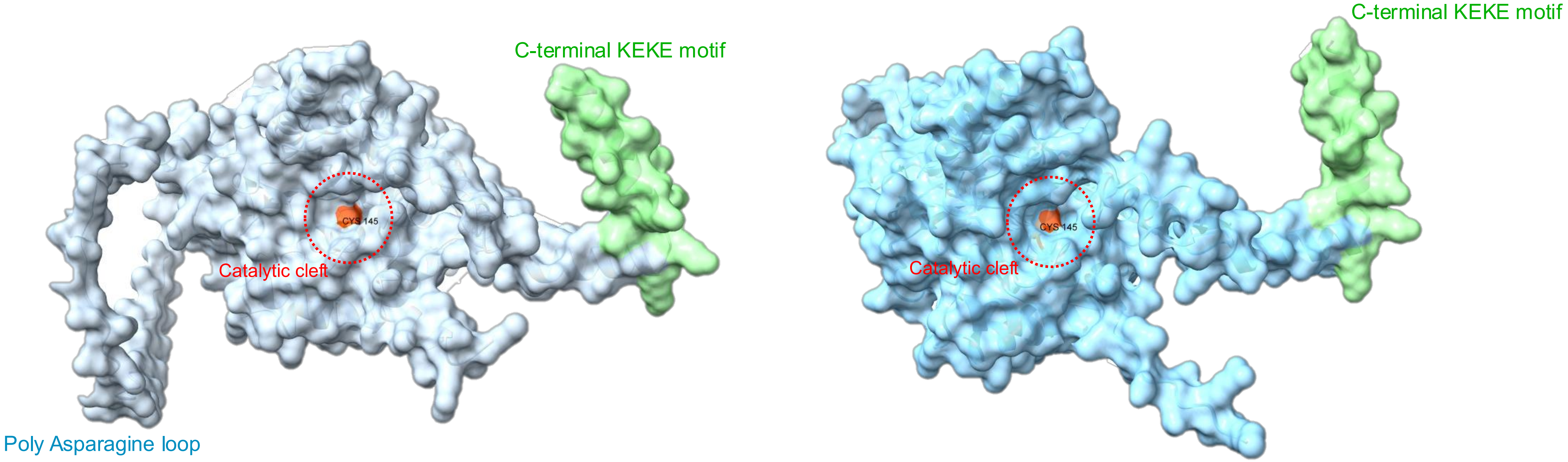

Supplementary Figure 2. Solvent-accessible surface area (ASA) representation of PfUCH37 wild type (left) and PfUCH37dN (right). The catalytic cysteine (C145), located at the core of the catalytic cleft, is highlighted in orange, while the C-terminal KEKE motif is shown in green. Truncation of the poly-asparagine region does not affect the accessibility of these critical regions. Structures generated by Aphafold3 and visualized by ChimeraX.

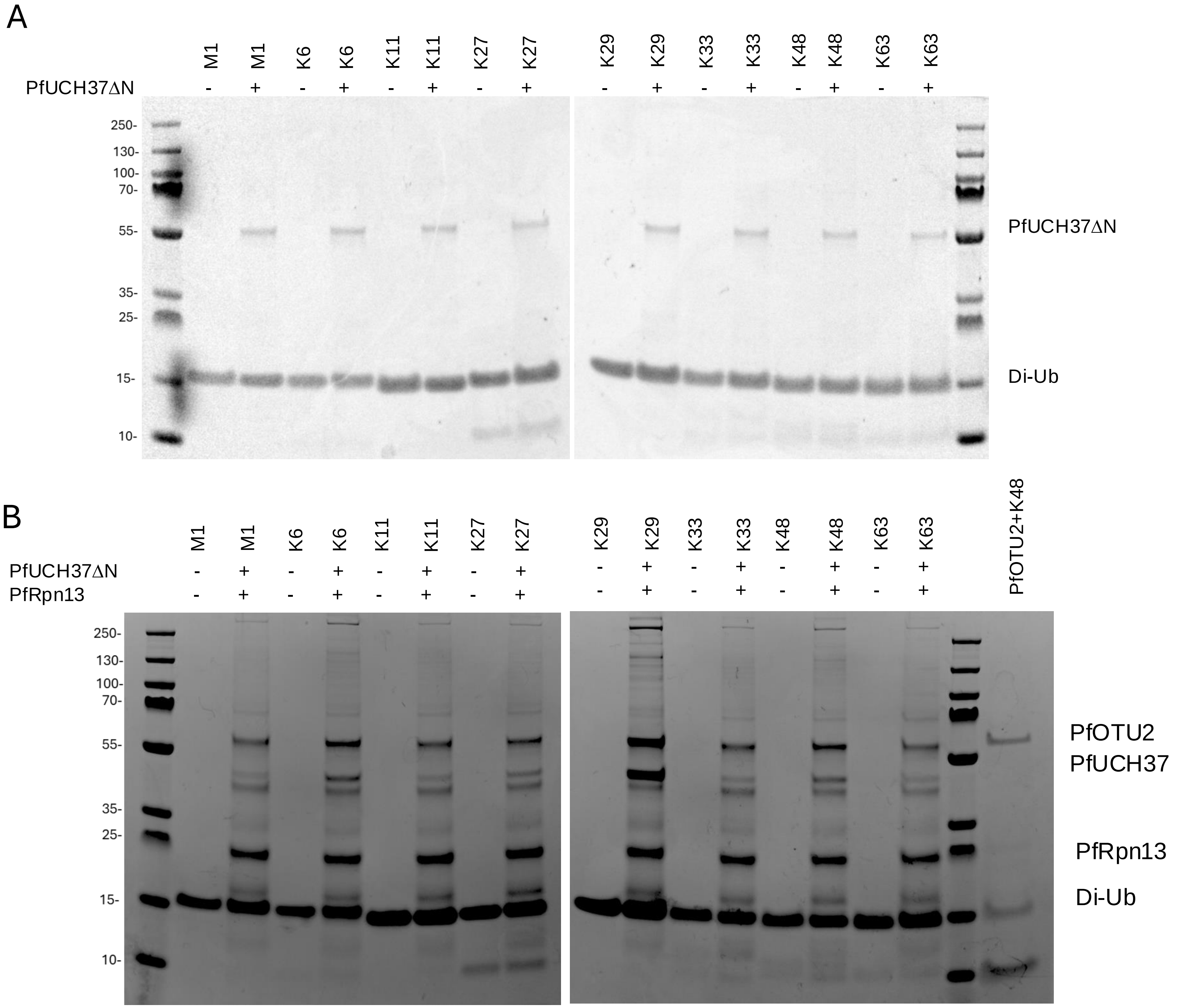

Supplementary Figure 3. Di-Ubiquitin Hydrolase Activity Assay. HA-UCH37dN and FLAG-PfRpn13 were expressed in HEK293 cells, purified using magnetic beads, and incubated with 1 µg Di-Ubiquitin in reaction buffer for 1 hour at 37°C. Reactions were terminated with reducing sample buffer, and proteins were analyzed via SDS-PAGE followed by Coomassie Blue staining.
A. PfUCH37 does not exhibit di-Ubiquitin hydrolysis activity toward Di-Ub in the absence of Rpn13.
B. PfUCH37 remains inactive in the presence of Rpn13. A *Plasmodium* deubiquitinating enzyme is used as a positive control (PfOTU2, last panel).
